# Choice of initial therapy

**DOI:** 10.7448/IAS.17.4.19488

**Published:** 2014-11-02

**Authors:** Manuel Battegay

**Affiliations:** Division of Infectious Diseases & Hospital Epidemiology, University Hospital Basel, Basel, Switzerland

## Abstract

Current international and national treatment guidelines such as EACS, BHIVA, DHHS or IAS update regularly recommendations on the choice of initial combination antiretroviral treatment (cART) regimens. Preferred cART regimens include a backbone with two nucleoside (nucleotide) reverse transcriptase inhibitors combined either with one non-nucleoside reverse transcriptase inhibitor or one ritonavir boosted protease inhibitor or more recently one integrase inhibitor. Response rates according to viral load measurements increased in recent years, in particular due to better tolerability. The choice of initial therapy is flexible and influenced by several factors such as height of viral load, genotypic resistance testing, CD4 cell count, co-morbidities, interactions, potential adverse events, (potential for) pregnancy, convenience, adherence, costs as well as physician's and patient's preferences. Diverse highly potent initial cART regimens exist. Following the many possibilities, the choice of a regimen is based on a mixture of evidence-informed data and individualized concepts, some of the latter only partly supported by strong evidence. For example, different perceptions and personal experiences exist about boosted protease inhibitors compared to non-nucleoside reverse transcriptase inhibitors or integrase inhibitors and vice versa which may influence the initial choice. This lecture will discuss choices of initial cART in view of international guidelines and the evidence for individualization of initial HIV therapy.

